# Common mental disorders in Brazilian adolescents: association with school characteristics, consumption of ultra-processed foods and waist-to-height ratio

**DOI:** 10.1590/0102-311XEN068423

**Published:** 2024-05-17

**Authors:** Lucia Helena Almeida Gratão, Thales Philipe Rodrigues da Silva, Luana Lara Rocha, Mariana Zogbi Jardim, Tatiana Resende Prado Rangel de Oliveira, Cristiane de Freitas Cunha, Larissa Loures Mendes

**Affiliations:** 1 Universidade Federal de Minas Gerais, Belo Horizonte, Brasil.; 2 Universidade Federal de São Paulo, São Paulo, Brasil.; 3 Pontifícia Universidade Católica de Minas Gerais, Belo Horizonte, Brasil.

**Keywords:** Adolescent Health, Mental Health, Food and Beverages, Saúde do Adolescente, Saúde Mental, Alimentos e Bebidas, Salud del Adolescente, Salud Mental, Alimentos y Bebidas

## Abstract

Half of all mental health problems diagnosed in adulthood have their onset before or during adolescence, especially common mental disorders (CMD). Thus, it is relevant to study the factors associated with these disorders. This study aimed to investigate the association of school characteristics, consumption of ultra-processed foods, and waist-to-height ratio with the presence of CMD in Brazilian adolescents. This is a school-based, cross-sectional study that analyzed data from 71,553 Brazilian adolescents aged 12-17 years. The prevalence of CMD in these adolescents was 17.1% (cut-off point 5 for the *General Health Questionnaire-12*). Associations were estimated using multilevel logistic models, with the presence of CMD as the dependent variable. The final model, adjusted for non-modifiable individual variables, modifiable individual variables and family characteristics, identified a positive association between private-funded schools (OR = 1.10; 95%CI: 1.07-1.14), advertisements for ultra-processed foods (OR = 1.13; 95%CI: 1.09-1.17), the second to fourth quartiles of ultra-processed food intake and waist-to-height ratio (OR = 2.26; 95%CI: 2.03-2.52). This study demonstrated that the private-funded schools , the presence of ultra-processed food advertisements, the consumption of ultra-processed food, and an increased waist-to-height ratio are risk factors for CMD in Brazilian adolescents.

## Resumo

Metade de todos os problemas de saúde mental diagnosticados na idade adulta têm seu início antes ou durante a adolescência, especialmente os transtornos mentais comuns (TMC). Desta maneira, é importante estudar os fatores associados a estes transtornos. Este estudo objetiva investigar a associação entre características escolares, consumo de alimentos ultraprocessados e razão cintura/estatura com a presença de TMC em adolescentes brasileiros. Trata-se de um estudo transversal de base escolar que analisou dados de 71.553 adolescentes brasileiros de 12-17 anos. A prevalência de TMC nesses adolescentes foi de 17,1% (ponto de corte 5 para o *Questionário de Saúde Geral* - GHQ-12). As associações foram estimadas por meio de modelos logísticos multiníveis, tendo como variável dependente a presença de TMC. O modelo final, ajustado para variáveis individuais não modificáveis, variáveis individuais modificáveis e características familiares, identificou uma associação positiva entre dependência administrativa privada (OR = 1,1; IC95%: 1,07-1,14), presença de propaganda de alimentos ultraprocessados (OR = 1,13; IC95%: 1,09-1,17), segundo a quarto quartis de consumo de alimentos ultraprocessados e razão cintura/estatura (OR = 2,26; IC95%: 2,03-2,52). Este estudo demonstrou que a dependência administrativa privada da escola, a presença de propagandas de alimentos ultraprocessados, o consumo de alimentos ultraprocessados e o aumento da razão cintura/estatura são fatores de risco para TMC em adolescentes brasileiros.

## Resumen

La mitad de todos los problemas de salud mental diagnosticados en la edad adulta empiezan antes o durante la adolescencia, sobre todo los trastornos mentales comunes (TMC). Así, es importante estudiar los factores asociados a estos trastornos. El objetivo de este estudio es investigar la asociación entre las características escolares, el consumo de alimentos ultraprocesados y la proporción cintura/estatura y la presencia de TMC en adolescentes brasileños. Se trata de un estudio transversal de base escolar que analizó datos de 71.553 adolescentes brasileños de 12 a 17 años. La prevalencia de TMC en estos adolescentes fue del 17,1% (punto de corte 5 para el *Cuestionario General de Salud* - GHQ-12). Se estimaron las asociaciones a través de modelos logísticos multinivel, con la presencia de TMC como variable dependiente. El modelo final, ajustado para variables individuales no modificables, variables individuales modificables y características familiares, identificó una asociación positiva entre la dependencia administrativa privada (OR = 1,10; IC95%: 1,07-1,14), la presencia de publicidad de alimentos ultraprocesados (OR = 1,13; IC95%: 1,09-1,17), segundo al cuarto cuartil de consumo de alimentos ultraprocesados y la proporción cintura/estatura (OR = 2,26; IC95%: 2,03-2,52). Este estudio demostró que la dependencia administrativa privada de la escuela, la presencia de publicidades de alimentos ultraprocesados, el consumo de alimentos ultraprocesados y el aumento de la proporción cintura/estatura son factores de riesgo para trastornos mentales comunes en adolescentes brasileños.

## Introduction

Adolescence is a transitional period between childhood and adulthood, during which individuals undergo profound physical, social, cognitive, and psychological changes ^1^. In this stage, individuals develop autonomy, self-control, interaction, and learning in society, which are important skills for strengthening mental health, both during this period and in subsequent stages of development ^2^.

According to the World Health Organization ^2^, half of all mental health problems diagnosed in adulthood have their onset before or during adolescence, especially common mental disorders (CMD). The term CMD refers to two main categories of diagnoses: anxiety and depressive disorders, and non-specific and somatic complaints, which may or may not be associated ^3^ ^,^ ^4^ ^,^ ^5^. These disorders are among the leading causes of illness in childhood and adolescence, increasing the risk of self-harm and suicide in this age group ^6^ ^,^ ^7^. In this sense, recognizing factors that may be associated with CMD could help guide action plans to mitigate these disorders in adolescents.

To better understand the factors associated with adolescents’ mental health, it is essential to observe the environments in which they live, such as schools. Some studies have shown that the school environment may be associated with the development of mental disorders ^8^ ^,^ ^9^. The school environment is where adolescents spend at least 20 hours per week - if enrolled in part-time education - and up to 40 hours per week - if enrolled in full-time education ^10^. In these spaces, besides educational activities, students receive or purchase meals, or both. In some cases, these places also feature food advertisements that direct the purchase and consumption of food. According to some studies conducted in Brazil, private schools have a more obesogenic food environment compared to public schools, due to the high availability, access to and presence of advertisements and ultra-processed foods ^11^, which are associated with overweight and chronic noncommunicable diseases in children and adolescents ^11^ ^,^ ^12^.

The scientific literature has explored the association between ultra-processed foods and mental health in adults ^12^ ^,^ ^13^ ^,^ ^14^ ^,^ ^15^ ^,^ ^16^ ^,^ ^17^ and adolescents ^18^ ^,^ ^19^. Studies have suggested that the consumption of ultra-processed foods may be associated with mental disorders ^13^ ^,^ ^14^ ^,^ ^15^ ^,^ ^16^ ^,^ ^17^ ^,^ ^18^ ^,^ ^19^. However, regardless of the design, these studies only considered the intake of ultra-processed foods, without considering other food environment factors. We believe that in addition to the consumption of ultra-processed foods, considered in isolation, some factors in the school environment, such as the type of school, the presence of advertising for ultra-processed foods and overweight, may be associated with CMD in Brazilian adolescents, as mental disorders have multifactorial causes ^20^, including environmental characteristics.

This study aimed to investigate the association between school characteristics (type of school funding and presence of ultra-processed foods advertisements) and individual characteristics (ultra-processed foods consumption and waist-to-height ratio - WHtR) related to food consumption and body adiposity with the presence of CMD in Brazilian adolescents.

## Materials and methods

### Design, sampling, and participants

The data used in this study were obtained from the *Brazilian Study of Cardiovascular Risk in Adolescents* (ERICA, acronym in Portuguese). ERICA was a cross-sectional, nationwide, school-based study with data collection carried out from March 2013 to December 2014. Its sample consisted of adolescents aged 12 to 17 years, of both sexes, enrolled in the last three years of middle school and the three years of high school, in public and private schools in Brazil. In addition to adolescents, school administrators were also interviewed ^21^.

The ERICA study included 273 Brazilian cities. To determine the number of eligible cities, the sampled population was divided into 32 geographic strata: each capital of the 27 Federative Units and five strata of metropolises with more than 100,000 inhabitants in each of the five macroregions of Brazil. After this geographic stratification, a selection of schools and classes in the eligible municipalities was carried out ^21^.

In the first stage, schools in each geographic stratum were selected with probability proportional to their size, which was considered equal to the ratio between the number of students in the eligible classes and the distance from the state capital. The selection was made after classifying the school records by location (urban or rural areas) and type of school funding (private or public). In total, 1,251 schools in 124 municipalities were selected ^21^.

In the second stage three classes from each school in the sample were selected with equal probability. Using Brazilian grade year as a proxy for age, 7th, 8th, and 9th grades of middle school and 10th, 11th and 12th grades of high school were considered eligible for selection. In each selected class, all students were invited to take part in the research, which consisted of interviews, anthropometric measurements and blood pressure measurements ^21^.

Detailed information on the sampling process, research protocol, participant selection and data collection can be found in studies previously published by the ERICA Study Committee ^21^ ^,^ ^22^ ^,^ ^23^.

### Instruments and data collection

ERICA included three questionnaires: one for adolescents, one for parents/educators and one for school administrators. For the analysis of this study, the questionnaire for adolescents was used, including a 24-hour dietary recall (24hR), anthropometric measurements (height and waist circumference) and the school questionnaire.

The questionnaire for adolescents consisted of 105 questions, with specific ones for each of the 11 thematic blocks, which consisted of sociodemographic characteristics, work and employment, physical activity, eating habits, smoking, alcohol consumption, reproductive health, oral health, reported morbidity, sleep duration, and mental health. The adolescents completed the questionnaire using the personal digital assistant (PDA, model LG GM750Q, https://www.lg.com/br/suporte/produto/lg-GM750Q.ABRATN) electronic device for data collection.

The school questionnaire consisted of 28 questions covering three thematic blocks: general school characteristics, physical structure and school food. The questionnaire was administered during an interview between the field researcher, using a PDA device, and the principal or another staff member of the school.

**Figure 1:**
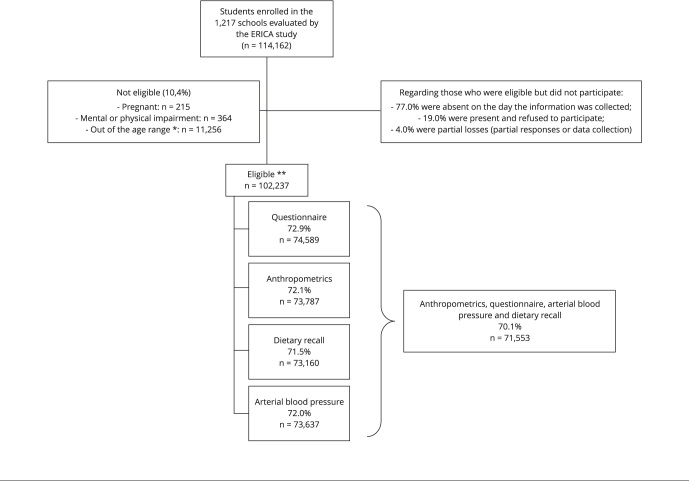
Flowchart of eligible adolescents and sample completeness regarding the information blocks and subsets. *Brazilian Study of Cardiovascular Risk in Adolescents* (ERICA), 2013-2014.

Source: Bloch et al. ^21^. * Younger than 12 and older than 17; ** Of the 102,327 eligible students, 23.7% did not answer any of the information blocks: questionnaire, anthropometrics, arterial blood pressure and 24-hour dietary recall.

The 74,953 eligible adolescents took part in the ERICA study. Of these, 74,589 completed the questionnaire for adolescents, 71,553 responded to the 24hR, 73,787 had their anthropometric measurements taken and 73,637 underwent blood pressure testing. For this study, only adolescents who participated in all of the steps mentioned above were considered, that is, 71,553 adolescents (Figure 1). According to the sensitivity analysis, this generated no significant difference in the final sample ^21^. Adolescents with some degree of disability that could affect the anthropometric assessment or prevent them from completing the questionnaire were excluded from the sample, as well as pregnant adolescents and the ones who were out of the age range (Figure 1).

### Dependent variable

To create the CMD variable, the *General Health Questionnaire* (GHQ-12), validated for use in adolescents ^24^, was included in the questionnaire for adolescents. The GHQ-12 is a widely used self-administered instrument that is known to be a reliable measure of mental health and assists in screening for psychiatric disorders in community and non-psychiatric clinical settings, using an index generated from individuals’ responses ^25^.

To screen for CMD in adolescents, the binary system with a shear point of 5 was considered - in other words, CMD was considered present if at least 5 of the 12 items were answered with one of the last two options of the questionnaire (“a little more than usual” or “much more than usual”). This cut-off point had a 86.7% sensitivity, a 88.9% specificity, a 71.2% positive predictive value and a 0.94 area under the receiver operating characteristics curve (ROC) ^26^.

### Independent variables

For the selection of independent variables, those related to schools and those associated with the consumption of ultra-processed foods and increased WHtR were tested: type of school funding (public or private) and presence of ultra-processed foods advertising (yes or no), consumption of ultra-processed foods in the previous 24 hours (quartiles of kcal/day) and WHtR (numerical variable).

To create the variable “presence of advertisements for ultra-processed foods”, advertisements for sweets, candy, lollipops, chocolate, sweet cookies, soft drinks, natural guarana, mate tea, other iced teas, guarana, isotonic, ice cream, popsicles and other ultra-processed foods were aggregated from the school questionnaire. Ultra-processed foods advertising was considered present if the school had at least one advertisement for these foods.

Data from the 24hR were used to determine ultra-processed foods intake. The 24hR data were collected during face-to-face interviews carried out by trained researchers. The Brasil-Nutri software (http://nebin.com.br/novosite/conteudo.php?id=4) was used to record food consumption data directly on the netbooks. The interview technique used was the multiple-pass method, which consists of a guided interview in five stages, to reduce underreporting of food consumption. The food database used in the research was developed by Brazilian Institute of Geography and Statistics (IBGE, acronym in Portuguese) in 2008-2009 ^27^ ^,^ ^28^.

After food weight was converted to grams, the dataset was linked to a nutritional composition table to calculate each adolescent’s energy intake. Foods were classified based on the degree of processing, as indicated by the NOVA food classification system ^29^. This system classifies all foods into the following four groups according to the nature, extent and purpose of the industrial processes they undergo: unprocessed or minimally processed foods; culinary ingredients; processed foods; and ultra-processed foods. Foods were categorized by two independent researchers and, in the event of disagreement, evaluated by a third expert researcher. For each adolescent, the total daily energy intake (kcal/day) was quantified and calculated in quartiles.

The WHtR was calculated by dividing the adolescents’ waist circumference by their height ^30^. Waist circumference was obtained using an anthropometric fiberglass tape (Sanny, https://www.sanny.com.br/) with a resolution in millimeters and a length of 1.50 meters. Height was obtained by averaging two consecutive measurements obtained with a portable and detachable stadiometer (Alturexata, http://www.alturexata.com.br/) with millimeter and field resolution of use up to 213 centimeters. Standardized procedures and training were used to ensure the quality of the information to be obtained both from the questionnaire and from direct measurements.

### Adjustment variables

The age of the adolescents was divided into the two following categories: 12-14 years and 15-17 years, in accordance with the classification used in other articles published with data from ERICA ^31^. For gender, the alternatives in the questionnaire for students were: female and male. The variable region of Brazil referred to five Brazilian macroregions: North; South; Central-West; Northeast; and Southeast.

The time of practice of weekly physical activity was categorized according to the cut points proposed by the *Brazilian National Survey of School Health* (PeNSE, acronym in Portuguese), in which students who did not practice physical activity in the reference period were considered inactive; those who practiced physical activity for 1-149 minutes were placed into the category “insufficiently active 1”; those who practiced physical activity for 150-299 minutes were placed into the category “insufficiently active 2”; and those who practiced for 300 minutes or more were categorized as active ^32^.

The variable “living with parents” referred to the following two categories: lives with both parents or only with the mother/father and does not live with either parent. The variable “work” was constructed using two variables based on the questions “did the student work without pay in the last year?” and “did the student work with pay in the last year?”, that is, the performance of paid and unpaid activities was considered work. Therefore, the categories of the variable considered for this study were: “does not work” and “works”.

To obtain the variable “mean sleep time”, the weighted mean between the usual duration, in hours, of sleep on weekdays and weekend days was calculated separately. Those who reported sleeping less than four hours and more than 14 hours were excluded for not meeting the usual parameters of sleep for adolescents.

To establish the socioeconomic status of the adolescents, it was decided to calculate a pattern of socioeconomic indicators (Supplementary Material. Table S1: https://cadernos.ensp.fiocruz.br/static//arquivo/suppl-e00068423_7936.pdf) by principal component analysis (PCA), consisting of the variables identified in the study by Ribeiro et al. ^33^, namely: the presence of employees in the residence; the number of residents per room; the number of bathrooms in the residence; and the number of refrigerators in the residence. The pattern of socioeconomic indicators generated by the PCA identified a single main component, with a contribution of 36.22% of the explained accumulated variation. The pattern was characterized by the presence of employees, fewer residents per room, more bathrooms, and more refrigerators in the residence (Supplementary Material. Table S1: https://cadernos.ensp.fiocruz.br/static//arquivo/suppl-e00068423_7936.pdf).

### Statistical analysis

Descriptive analysis included the calculation of absolute and relative frequencies for categorical variables, in addition to measures of central tendency. The chi-square test and t-test were used to compare proportions between variables.

The association between school characteristics, individual characteristics and the presence of CMD was estimated using multilevel logistic models, with the presence of CMD as the dependent variable. The inclusion of adjustment variables followed a hierarchical pattern (including only the independent variables, then the adjustment variables that were non-modifiable characteristics, and, lastly, the modifiable characteristics).

Thus, four models were proposed: (i) null model (M0), estimating the random effect of the intercept; (ii) model 1 (M1), containing the independent variables (type of school funding [public/private], ultra-processed foods advertising at school [no/yes], ultra-processed foods consumption [quartile of total kcal of ultra-processed foods consumed per day] and WHtR [numerical variable]), and non-modifiable adjustment variables (sex [female/male], age [12-14 and 15-17] and race/color [white/black/mixed-race/yellow/Indigenous]); (iii) model 2 (M2), containing the variables of M1 in addition to modifiable individual variables (work activities by the adolescent, total daily energy intake [numerical variable], mean sleep time [numerical variable], physical activity [inactive, insufficiently active 1, insufficiently active 2, active]); and (iv) model 3 (M3), containing the variables of M2 in addition to adjustment variables related to family characteristics (living with parents [both parents or only the mother/father] or not), the pattern of socioeconomic characteristics (terciles of pattern) and the region of residence (North, South, Central-West, Southeast, and Northeast).

The variance partition coefficient (VPC) was quantified to verify the proportion of total variance attributed to the schools. The assessment of the models was done by comparing the values of Akaike’s information criterion (AIC), in which a decrease in the AIC value indicates a better fit of the model to the response variable. At the end of the modeling, the variance reduction was calculated to verify the final fit.

The statistical software for professionals Stata, version 14.0 for Mac (https://www.stata.com), package was used. For the multilevel models, the “gllamm” command was used, allowing for non-independent data and multilevel analyses with the inclusion of sample weights for complex samples. The aggregation unit used was the adolescents’ school. A 5% significance level was used for all analyses.

### Ethical aspects

This report was approved by the Institutional Review Board of the Institute of Collective Health Studies of the Federal University of Rio de Janeiro (IESC/UFRJ, acronym in Portuguese), which is part of the central coordination of the report (approval n. 45/2008), and of each Brazil’s Federative Units. Written informed consent was obtained from all subjects, their parents and legal guardian(s) in two copies, one of which remained in the possession of the research subjects. The adolescents also signed a written assent form.

## Results

### Sample characteristics

Data from 71,553 Brazilian adolescents were evaluated. The prevalence of CMD in these adolescents was 17.1% (cut-off point 5 for the GHQ-12). Table 1 shows the characterization of the adolescents studied, most of whom were male (50.21%); aged 12 to 13 years (35.1%); mixed-race (48.83%); belonged to the first tercile of the pattern “socioeconomic status”, which corresponds to those with better socioeconomic conditions (46.16%); resided in the Southeast Region (50.78%); and studied in public schools (83.61%) located in the urban area (96.1%). Of the adolescents studied, 26.07% performed some work activity, 36.85% lived with their parents or only with their mother, and 5.88% did not live with either parent.

**Table 1:** Characteristics of adolescents enrolled in schools in Brazilian capitals stratified by the presence of common mental disorders (CMD). *Brazilian Study of Cardiovascular Risk in Adolescents* (ERICA), 2013-2014, (n = 71,553).

Parameter	CMD				
	Total sample (n) *	Total sample (%) **	CMD- (%)	CMD+ (%)	p-value ***
Individual and family characteristics					
Sex					
Female	39,690	49.79	46.05	67.92	< 0.001
Male	31,863	50.21	53.95	32.08	
Age (years)					
12-14	32,840	52.70	54.14	45.74	< 0.001
15-17	38,713	47.30	45.86	54.26	
Region of residence					
Central-West	9,331	7.67	7.60	7.98	0.7908
Northeast	22,205	21.34	21.41	20.97	
North	14,494	8.43	8.38	8.67	
Southeast	16,434	50.78	50.87	50.37	
South	9,089	11.78	11.74	12.01	
Work activities by the adolescent					
No	54,190	73.97	74.93	69.32	< 0.001
Yes	17,363	26.03	25.07	30.68	
Pattern of socioeconomic indicators (tertiles) ^#^					
First	31,609	46.26	46.08	47.11	0.2841
Second	24,864	35.04	35.32	33.68	
Third	14,349	18.70	18.60	19.21	
Lives with parents					
Both parents or only with the mother/father	66,634	92.12	94.56	92.01	< 0.001
None of the parents	4,919	5.88	5.44	7.99	
Practice of physical activity					
Inactive	13,047	16.72	15.59	22.20	< 0.001
Insufficiently active 1	10,148	14.19	14.21	14.13	
Insufficiently active 2	9,686	14.05	14.50	11.88	
Active	38,672	55.04	55.71	51.79	
Type of school funding					
Public	56,703	83.61	83.70	83.19	0.4025
Private	14,850	16.39	16.30	16.81	
Ultra-processed food advertisements at school					
No	62,201	95.35	95.41	95.05	0.1422
Yes	6,024	4.65	4.59	4.95	
		General mean (SD)	CMD mean (SD)	CMD+ mean (SD)	p-value ^##^
Individual variable ^##^					
Daily energy intake	71,553	2,286.78 (20.21)	2,293.01 (22.07)	2,256.34 (23.89)	< 0.001
Ultra-processed foods consumption (quartiles)	71,553				
First	17,910	87.13 (72.77)	86.69 (72.89)	89.18 (72.18)	0.080
Second	17,867	353.65 (84.39)	353.67 (84.48)	354.65 (83.98)	0.554
Third	17,888	725.38 (141.60)	724.24 (141.48)	730.30 (142.03)	0.026
Fourth	17,888	1,606.40 (725.11)	1,597.55 (713.56)	1,644.51 (771.87)	< 0.001
Waist-to-height ratio	71,553	0.43 (0.001)	0.43 (0.001)	0.44 (0.001)	< 0.001
Mean sleep time	71,553	8.41 (0.31)	8.46 (0.31)	8.16 (0.39)	< 0.001

SD: standard deviation.

Note: values in bold indicate statistical significance (p < 0.05).

* Sample number without using sample weight;

** Frequency of the sample using sample weight, that can be extrapolated to the Brazilian population;

*** Chi-square test;

^#^ The pattern of socioeconomic indicators was characterized by a higher number of employees in the household, a lower number of residents per room, and a higher number of bathrooms and of refrigerators in the household;

^##^ T-test.

The presence of CMD was more frequent and statistically significant among female adolescents (67.98%), aged 14-15 years (36.05%), who did not work (69.38%), had mothers with an education level up to complete middle school (33.55%) and who lived with both parents (51.46%) (Table 1). It was also observed that 26.34% of the adolescents with CMD had a WHtR of 0.44±0.001. The mean energy intake was 2,352.72±26.10 kcal/day (Table 1), a higher value than that observed in adolescents without CMD.

### Association between school characteristics and CMD

Table 2 shows the multilevel logistic regression models, with the presence of CMD as the outcome variable and the type of school funding, ultra-processed foods consumption, presence of ultra-processed foods advertising at school and WHtR as independent variables.

**Table 2:** Multilevel logistic regression models for ultra-processed foods consumption, waist-to-height ratio, and school environment variables associated with common mental disorders (CMD) in adolescents enrolled in Brazilian schools. *Brazilian Study of Cardiovascular Risk in Adolescents* (ERICA), 2013-2014 (n = 71,553).

Parameter	Null model	Model 1 *		Model 2 **		Model 3 ***	
	OR (95%CI)	OR (95%CI)	p-value	OR (95%CI)	p-value	OR (95%CI)	p-value
School characteristics							
Type of school funding							
Public	-	Reference	< 0.001	Reference	< 0.001	Reference	< 0.001
Private	-	1.11 (1.08-1.14)		1.12 (1.09-1.15)		1.11 (1.08-1.15)	
Ultra-processed foods advertising							
No	-	Reference	< 0.001	Reference	< 0.001	Reference	< 0.001
Yes	-	1.16 (1.13-1.20)		1.12 (1.09-1.16)		1.10 (1.06-1.14)	
Individual characteristics of adolescents							
Ultra-processed foods intake (quartiles)							
First	-	Reference	< 0.001	Reference	< 0.001	Reference	< 0.001
Second	-	1.10 (1.08-1.12)		1.08 (1.06-1.10)		1.08 (1.06-1.10)	
Third	-	1.07 (1.05-1.09)	< 0.001	1.03 (1.01-1.05)	< 0.001	1.04 (1.02-1.05)	< 0.001
Fourth	-	1.17 (1.15-1.19)	< 0.001	1.19 (1.17-1.22)	< 0.001	1.20 (1.18-1.22)	< 0.001
Waist-to-height ratio	-	2.88 (2.61-3.18)	< 0.001	2.32 (2.08-2.58)	< 0.001	2.16 (1.94-2.41)	< 0.001
Fixed effects							
Intercept	0.20 (0.205-0.209)	0.13 (0.13-0.14)	< 0.001	0.44 (0.41-0.47)	< 0.001	0.44 (0.41-0.47)	< 0.001
Random effects							
Variance	0.18 (0.003)	0.15 (0.0034)		0.17 (0.0040)		0.17 (0.0039)	
Variance partition coefficient	0.0518	0.0461		0.0505		0.0498	
AIC	1,009,926	893,945.8		779,477.8		769,378.3	

95%CI: 95% confidence interval; AIC: Akaike’s information criterion; OR: odds ratio.

* Model 1 was adjusted for: sex (female/male), age (12-14 and 15-17);

** Model 2 was adjusted for Model 1 adjustment variables plus: work activities by the adolescent (yes/no); total kilocalories consumed in the previous 24 hours (numerical variable); mean sleep time (numerical variable); and physical activity (inactive, insufficiently active 1, insufficiently active 2, active);

*** Model 3 was adjusted for Model 2 adjustment variables plus: living with parents (both parents or only with the mother/father, or with none of the parents); terciles of the pattern of socioeconomic indicators (terciles of pattern); and region of residence (North, South, Central-West, Southeast, and Northeast).

Table 2 shows the M0. The intercept variation (0.20; 95%CI: 0.205-0.209) of M0 showed that the presence of CMD in adolescents differed between schools (p < 0.001). The variance partition coefficient (VPC) was 0.0518, that is, approximately 5.18% of the total variance was attributed to the characteristics of the schools of the adolescents.

M1 was adjusted only for sex, age, and race/color, which are factors that cannot be modified by the individual. It is possible to observe that there was a positive association between the private administration of the school (OR = 1.11; 95%CI: 1.08-1.14) and the presence of ultra-processed foods advertising in the school environment (OR = 1.16; 95%CI 1.13-1.20) with the presence of CMD.

In M2, with the inclusion of modifiable factors such as work activities by adolescents, total kilocalories consumed in the previous 24 hours, mean sleep time, and physical activity, a reduction in the odds ratio values for the school environment variables was observed. However, the type of funding of the school (OR = 1.12; 95%CI: 1.09-1.15) and the presence of ultra-processed foods advertising in the school (OR = 1.12; 95%CI: 1.09-1.16) remained directly associated with the presence of CMD.

M3, additionally adjusted for family characteristics, such as living with parents, the pattern of socioeconomic indicators and region of residence, maintained the behavior observed in M2, reducing the odds ratio of school environment variables, but still maintaining their association with the presence of CMD. There was a positive association between the private-funded schools (OR = 1.11; 95%CI: 1.08-1.15) and the presence of ultra-processed foods advertising in the school (OR = 1.10; 95%CI: 1.06-1.14).

### Association between ultra-processed foods consumption, WHtR and CMD

Comparing M1, M2 and M3, it can be observed that there was an increase in ultra-processed foods consumption in the fourth quartile, that is, among those who consumed more kilocalories from ultra-processed foods (M1: OR = 1.17, 95%CI: 1.15-1.19; M2: OR = 1.19, 95%CI: 1.17-1.22; M3: OR = 1.20, 95%CI: 1.18-1.22).

At the same time, the WHtR decreased in magnitude, as observed by the reduced odds ratio in M2 and M3 in relation to M1. However, in all of them, there was still a positive association with the outcome (M1: OR = 2.88, 95%CI: 2.61-3.18; M2: OR = 2.32, 95%CI: 2.08-2.58; and M3: OR = 2.16, 95%CI: 1.94-2.41).

## Discussion

This is the first study to identify an association between ultra-processed foods consumption, body adiposity, and characteristics of the school environment with the presence of CMD in adolescents. A positive association of the private administration of schools, ultra-processed foods advertising in schools, ultra-processed foods consumption and increased WHtR with the presence of CMD in Brazilian adolescents was found.

In addition, Hecht et al. ^34^ set out to investigate whether adults aged over 18 years who consumed ultra-processed foods had more symptoms related to mental health. They found that individuals with high ultra-processed foods consumption were significantly more likely to report depression and anxiety and to have worse mental health. Other studies have found a similar association ^35^ ^,^ ^36^ ^,^ ^37^. The physiological mechanisms associated with these events are not yet known; however, it has been hypothesized that industrial additives used for preservation, odorization, and coloring can modify the neuronal mitochondrial function by various metabolic pathways ^38^. The consumption of unhealthy foods has also been linked to inflammatory processes, nutrient and neurotransmitter defficiencies ^39^ and increased likelihood of central nervous system demyelination ^40^, as well as changes in the gut-brain axis, leading to changes in the production of neurotransmitters ^41^.

However, in the case of our study, the possibility that the presence of CMD can lead to a worsening of the quality of food choices cannot be ruled out, as there is a possibility that emotions regulate eating, just as eating can regulate emotions ^42^. Keck et al. ^43^, for example, in a study with 225 college students, observed that symptoms of depression were a greater risk factor for poor nutrition.

The association between parameters of body adiposity and the presence of CMD in adolescents has been found in this study and by other researchers. Our results showed that an increased WHtR may be associated with the presence of CMD in adolescents, as the WHtR is strongly correlated with visceral fat ^44^. Scott et al. ^45^, in a cross-sectional study of people aged over 16 years in New Zealand, found an association between obesity, depressive disorder and anxiety disorder. Lewis-de-Los-Angelis & Richard ^46^ found that a history of depression was associated with a higher WHtR in individuals from the United States aged 9 and 10 years WHtR. In addition, girls with a history of depression were found to be more likely to have an elevated WHtR.

Therefore, an increased WHtR is associated with CMD in adolescents. In another study with adults aged from 20-89 years old ^47^, the authors found that an increase in the waist-hip ratio was associated with an increase in the prevalence of anxiety and depression. Notably, there is a possibility of reverse causality when referring to the association between WHtR and CMD, given the study design, and also considering that the literature has shown a bidirectional association between body adiposity and mental health outcomes.

This leads us to believe that the neural mechanisms associated with the consumption of ultra-processed foods ^34^ ^,^ ^35^ and increased WHtR ^47^ may lead to adolescents having a greater chance of developing CMD, even during adolescence. For us, based on our results and on those of published studies, the advertising and sale of ultra-processed foods in private schools, favoring greater consumption of these foods ^48^ ^,^ ^49^ and increased body adiposity, increase the risk of CMD in adolescents.

In this regard, Carmo et al. ^50^ also found crucial data in a cross-sectional study with 1,427 public and private schools in Brazil, reporting that at least 76.1% of private schools marketed some ultra-processed foods. It is also known that the presence of unhealthy foods in the school environment is associated with higher consumption of these items by students ^51^ ^,^ ^52^ ^,^ ^53^ ^,^ ^54^. Rocha et al. ^51^ found that the caloric contribution of ultra-processed foods to the total kilocalories consumed by adolescents was significantly higher in those who studied in private schools.

Unlike public schools, private schools are profit-driven institutions, regulated by the Brazilian Ministry of Education only in terms of educational features ^55^. This means that these institutions do not have nationwide regulations on the advertising and marketing of foods and beverages on their premises, and it is up to them to determine how these items will be made available in the school environment. The food environment of private schools is characterized by the sale of ultra-processed foods and beverages inside and around their premises, in addition to the presence of food advertising ^50^ ^,^ ^56^ ^,^ ^57^ ^,^ ^58^ ^,^ ^59^.

A school environment is a privileged place for health and nutrition interventions, but when it is characterized as an obesogenic environment, there is a risk of immediate and long-term negative effects on the health of children and adolescents, especially concerning healthy habits and behaviors ^2^ ^,^ ^60^. In this sense, it may contribute to an increase in the prevalence of obesity ^61^ and is also a risk factor for CMD in adolescents.

This study has some limitations, such as the use of a 24hR of only one day to construct the variable identifying ultra-processed foods consumption, which may imply a consumption that does not correspond to that of the adolescents evaluated and the possibility of recall bias and individual attrition. To ensure that the data from this recall would be collected in the best possible way, the multiple-pass interview technique was used ^62^. The GHQ-12 ^63^, although validated for use in adolescents by French & Tait ^24^, may be subject to recall bias and divergent responses, in addition to the possibility of underestimating the cases of adolescents who are treated for mental illness with medication that reduces the symptoms of anxiety and depression, in which case this adolescent, even if diagnosed, may not be identified by the GHQ. Lastly, it is not possible to state that the ultra-processed foods consumption came from meals eaten in the school environment. We emphasize that there is no way to infer causality in this study, because it is a cross-sectional study. This design measures everything at the same time, with no way to define the temporality between risk factors and development. It is important to clarify that the factors studied can only be risk markers.

Despite its limitations, this investigation used the ERICA Study database, which was carefully constructed, as well as all the stages of the study, from sampling to data collection, with the more than 71,000 adolescents evaluated, representative of the adolescent population.

## Conclusions

In this study, it was possible to observe that the type of school funding, the presence of ultra-processed foods advertising, ultra-processed foods consumption and increased WHtR are risk factors for CMD in Brazilian adolescents.

This study showed the importance of the school environment as a health-promoting place and how characteristics of this environment can contribute to the presence of CMD in adolescents. Due to the study design, it was not possible to determine causal relationships, leaving gaps in how the food environment of private schools could exert this relationship with mental health in this age group.
